# Neurodiversity in mental simulation: conceptual but not visual imagery priming modulates perception across the imagery vividness spectrum

**DOI:** 10.1038/s41598-025-05100-2

**Published:** 2025-07-01

**Authors:** Ágnes Welker, Orsolya Pető-Plaszkó, Luca Verebélyi, Ferenc Gombos, István Winkler, Ilona Kovács

**Affiliations:** 1 HUN-REN Research Centre for Natural Sciences, Budapest, Hungary; 2 Semmelweis University Doctoral School, Budapest, Hungary; 3 Pázmány Péter Catholic University, Budapest, Hungary; 4 HUN-REN-ELTE-PPKE Adolescent Development Research Group, Budapest, Hungary; 5 Faculty of Education and Psychology, Eötvös Loránd University, Budapest, Hungary

**Keywords:** Neurodiversity, Mental simulation, Visual imagery, Binocular rivalry, Priming, Neuroaffirmative, Human behaviour, Consciousness, Perception

## Abstract

Mental simulation—the ability to internally model sensory, conceptual, or future events—may include mental imagery as a component, with considerable individual variability in its vividness and dependence on sensory detail. While self-reports have been widely used to assess imagery, they are subjective and prone to bias. Among more objective methods, imagery priming in binocular rivalry has been employed to investigate the influence of mental imagery on perception, but findings have been ambiguous. Here, we introduce a no-report version of the task, using eye-tracking-based optokinetic nystagmus assessment to provide a more reliable measure of perceptual shifts. In addition to visual imagery priming, we introduce conceptual priming, which does not rely on sensory imagery but engages abstract representations. In visual imagery priming, perceptual modulation correlated with self-reported vividness, and participants with low vividness did not show modulatory effects. However, in conceptual priming, effects were observed across the entire vividness spectrum, demonstrating that both concrete sensory-based and abstract conceptual representations can influence perception. These findings challenge purely sensory accounts of mental imagery. We propose avoiding deficit-based terms such as “aphantasia” and advocate for a neuroaffirmative perspective on mental simulation diversity.

**Supplementary Information:**

The online version contains supplementary material available at 10.1038/s41598-025-05100-2.

## Introduction

Neurodiversity underscores the natural variability in cognitive and neural functioning, framing differences in perception, memory, and mental simulation as expressions of normal human diversity rather than deficits. One such dimension of cognitive variation is visual mental imagery, which spans a spectrum from hyperphantasia^1,2^ (extremely vivid imagery) to aphantasia^1–3^ or hypophantasia^2,4^ (a complete absence or a reduced level of visual imagery respectively). To reflect a neuroaffirmative approach, we suggest replacing these traditional, deficit-based terms with a model that recognises distinct mental simulation styles. This approach is largely based on our results presented below, challenging the assumed exclusive link between visual imagery and sensory perception^5–7^.

Mental simulation refers to the internal rehearsal or modeling of events, actions, or scenarios, it may involve not only sensory experiences, but also conceptual, emotional, motor, or abstract processes^8^. Mental imagery, by contrast, is specifically a recreation of sensory-like experience in the mind without direct input from the senses^9^. It most often refers to visual imagery, but also includes auditory, olfactory, gustatory, and tactile imagery. Visual imagery, therefore, is a type of mental simulation—one that relies heavily on sensory-like representations.

Previous research has demonstrated that visual imagery priming can modulate perception in people with neurotypical levels of imagery vividness and this effect is reduced in those with aphantasia^5–7,10,11^. This raises an important question: does the lack of effectiveness of visual imagery priming in individuals with aphantasia or hypophantasia indicate a fundamental limitation in modulating sensory perception by internal processes? Or perhaps individuals with lower visual imagery vividness might engage alternative cognitive strategies when specifically asked to employ those? To address this issue, we explore alternative methods of inducing mental simulation and assess their effectiveness in modulating visual perception.

The above-mentioned results with visual imagery priming^5–7^ rely on the fact that ambiguous visual stimulation through the two eyes results in the bistability of conscious perception under Binocular Rivalry (BR). BR refers to the perceptual shifts that occur when each eye is presented with a distinct, conflicting image, causing the brain to alternate between the two, as it cannot process both simultaneously^12,13^. Based on the assumed functional interaction between visual imagery and sensory perception^14–18^, it has been hypothesized that visual imagery may affect the perceptual dominance rates in binocular rivalry^7^. The strength of this influence depended on the self-reported levels of imagery vividness^5–7^, suggesting a dynamic relationship between visual imagery and visual perception.

However, other studies have failed to replicate these results, finding no significant relationship between self-reported imagery vividness and binocular rivalry^19,20^. More recently, it has been concluded that while imagery priming can enhance sensitivity to the primed stimulus in BR tasks on a trial-by-trial basis (linked to reported vividness), the priming effect does not correlate with individual differences in subjective imagery vividness across participants^21^. The reliability of self-reported BR tasks used in previous studies has also been questioned^21^, highlighting the need for more robust objective measures of rivalry alternations. Here, we propose a no-report version of the BR approach as a methodological solution to this problem.

**Fig. 1:**
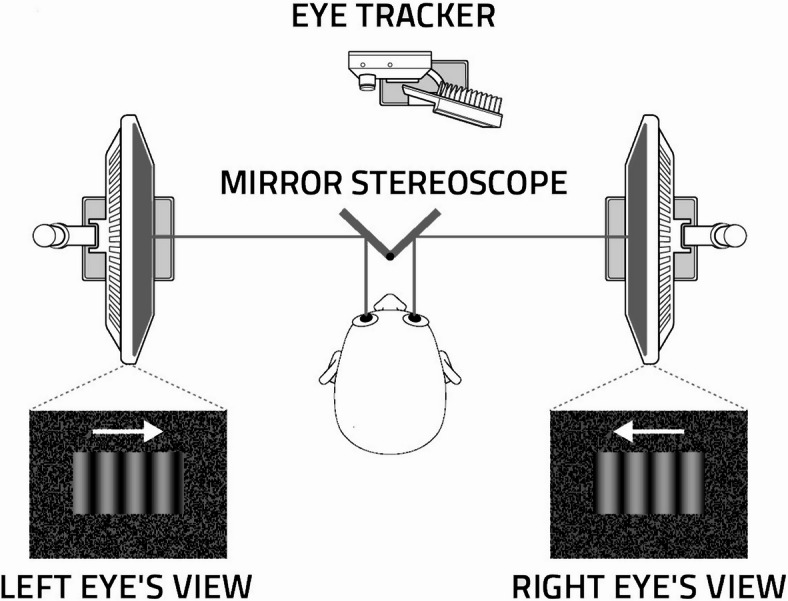
The Binocular Rivalry—Optokinetic Nystagmus (BR-OKN) No-Report Paradigm (adapted from^22–24^). A cold-mirror stereoscope provides separate views of the left and right eyes. Eye-movements are recorded throughout the stimulation. Rivalry is induced by sinusoidal gratings moving in opposite directions. Perceived motion direction and spontaneous reversals are reflected in oculomotor behaviour: the smooth pursuit phase of optokinetic nystagmus (OKN) corresponds to the dominant direction of motion. This eliminates the need for manual responses, such as button presses.

We have been using a no-report version of the BR paradigm complemented with eye-tracking based on optokinetic nystagmus (OKN), in which participants observe binocularly rivalrous gratings moving in opposite directions while their eye movements are monitored^22–24^ (see Fig. 1. and the Methods section). Perceptual changes in the stimulus, including spontaneous reversals in perceptual dominance, are reflected by the emergence of saccadic eye movements associated with OKN. This approach eliminates the need for subjects to manually indicate their perceived direction through button presses, allowing for precise and objective detection of shifts occurring during rivalry^22–24^.

**Fig. 2:**
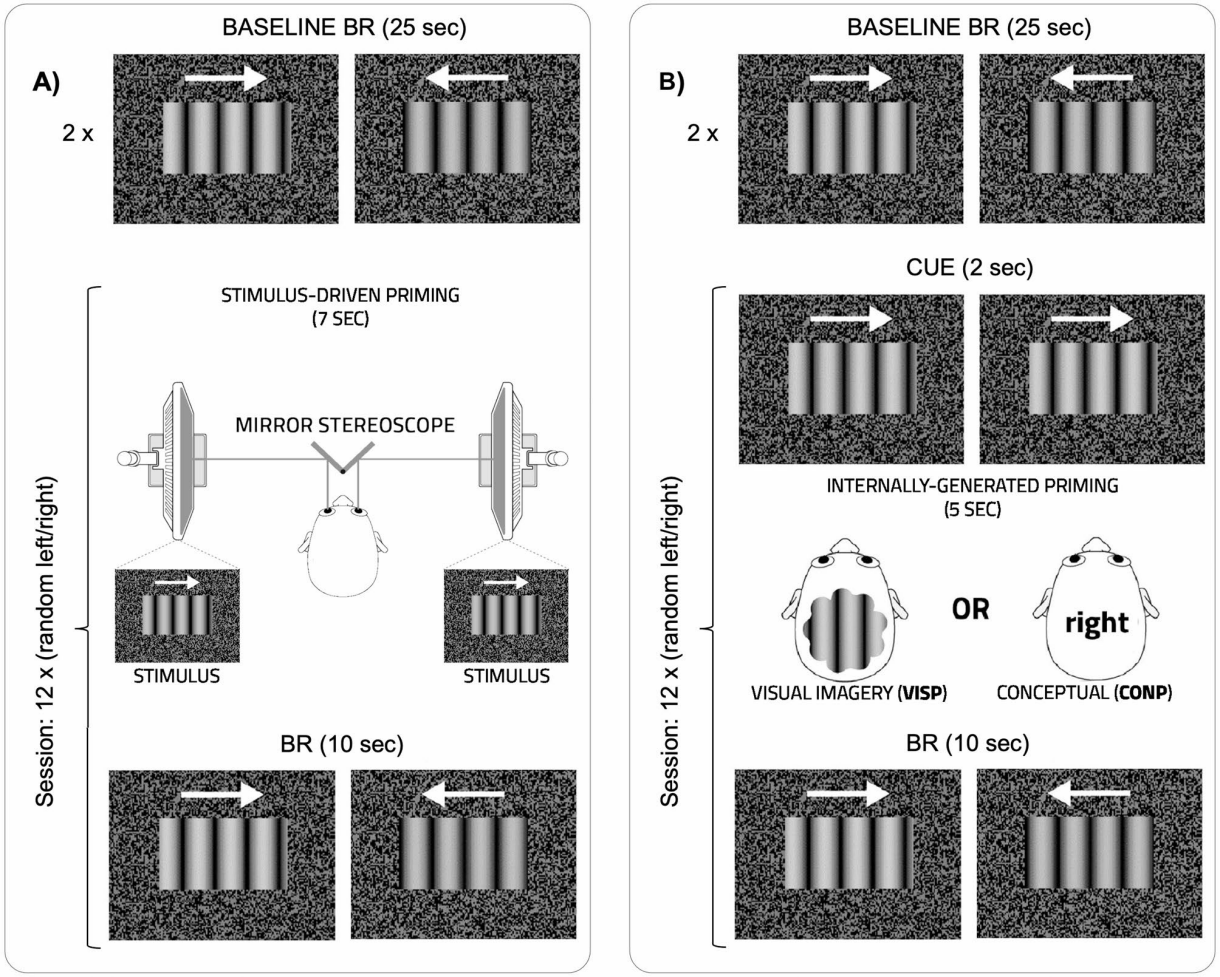
The Priming Conditions. Binocular rivalry (BR) alternations were assessed through repeated 25-s baseline measurements preceding each priming condition. After priming, a 10-s BR stimulation phase served as a test condition to evaluate the modulatory effect of priming. (**a**) Stimulus Driven Priming (SDP): After the baseline rivalry stimulus, each eye was presented with gratings moving in the same direction for 7 s. (**b**) Internally-Generated Priming: After a 2-s cue in which both eyes viewed gratings moving in the same direction, participants engaged in one of two priming tasks. Visual Imagery Priming (VISP): Participants were instructed to generate a mental image of the cued stimulus. Conceptual Priming (CONP): Participants were asked to think about the movement direction without forming a visual mental image.

To induce mental simulation of movement direction and assess its effectiveness in modulating visual perception, we implemented a priming task (see Fig. 2). A total of 119 participants, screened for neurological, psychiatric and neurodevelopmental disorders were categorised based on their Vividness of Visual Imagery Questionnaire (VVIQ^8^) scores (hypophantasia VVIQ < 48; neurotypical imagery 48 ≤ VVIQ < 75; hyperphantasia VVIQ ≥ 75), and asked to participate in the priming task. Stimulus Driven Priming (SDP)—unambiguous grating moving in one direction for the entire time of stimulation—(Fig. 2a) served as a reference for Internally-Generated Priming (Fig. 2b). In the SDP condition, it is expected that the typically observed inhibitory negative priming effect^6,20^ will occur across the entire spectrum of imagery vividness.

There were two types of Internally-Generated Priming blocks: Visual Imagery Priming (VISP) and Conceptual Priming (CONP). The VISP condition closely resembles previously employed versions of imagery priming in BR^5–7^. After receiving a cue (e.g., a grating stimulus moving to the right), participants were instructed to visually imagine that grating for a few seconds before being presented with a binocularly rivalrous stimulus. Based on previous findings^5–7^, we expected that only the hypophantasic group would fail to exhibit a positive priming effect.

As an alternative to visual imagery, we also included a CONP condition, in which participants were asked to think about the prime direction without forming an internal visual image. We hypothesized that, in this novel condition, even hypophantasic participants might activate internal representations related to the primed direction. Though these representations may be more abstract, they could still be sufficient to modulate perceptual alternations in BR.

We also hypothesized that there might be a trade-off between the effective activation of concrete (visual/sensory/episodic) versus more abstract (conceptual/semantic/linguistic) representations in mental simulation. In this case, we would expect visual imagery priming to correlate positively with self-reported imagery vividness, and conceptual priming would correlate negatively. This hypothesis was initially informed by pilot data, which suggested a negative correlation between self-reported vividness and conceptual priming. While this led us to speculate that individuals with vivid imagery might rely less on non-visual strategies, we acknowledge that high visual vividness does not necessarily preclude effective conceptual processing.

## Results

The Ratio of Prime Direction (RPD) was calculated as the proportion of eye movements in the direction of the primed grating during the first 1.5 s of unmixed perception (see Methods/Fig. 6). Mixed percept refers to periods of perceptual transition, where gaze velocity does not clearly indicate a dominant percept. This value served as the basis for our subsequent analyses, allowing us to examine differences across conditions and imagery groups. The complete statistical test results, including reliability analyses and the examination of gender differences (for which no significant results were found), are available in tabular form in the supplementary material. Reliability was assessed by repeating the full priming paradigm with 48 participants after a 10-min break, using a different condition order (CONP, SDP, VISP). Differences between sessions were analyzed using one-sample t-tests and Pearson correlations. No significant difference was found between the first and second sessions in any condition VISP (*p* = 0.819), CONP (*p* = 0.816), SDP (*p* = 0.605), indicating stable performance across sessions. Pearson correlations showed moderate to strong test–retest reliability VISP (r = 0.460, *p* < 0.001), CONP (r = 0.706, *p* < 0.001), SDP (r = 0.759, *p* < 0.001). These results support the internal reliability and temporal stability of the RPD measure in all three priming conditions.

### Perceptual modulation at the overall population level

One-sided one-sample t-tests were conducted to determine whether the RPD differed significantly from the baseline value of 0.5 across all conditions. Results indicated that in the VISP condition, RPD was significantly greater than 0.5 (t(87) = 2.868, *p* = 0.003, one-tailed, Cohen’s d = 0.306). Similarly, in the CONP condition, RPD was also significantly greater than 0.5 (t(79) = 3.380, *p* < 0.001, one-tailed, Cohen’s d = 0.378). In contrast, the SDP condition showed a significant decrease below 0.5 (t(88) = − 5.745, *p* < 0.001, one-tailed, Cohen’s d = − 0.609). These results suggest that all three priming conditions effectively modulated perception at the overall population level.

### Correlation between perceptual modulation and self-reported vividness

**Fig. 3:**
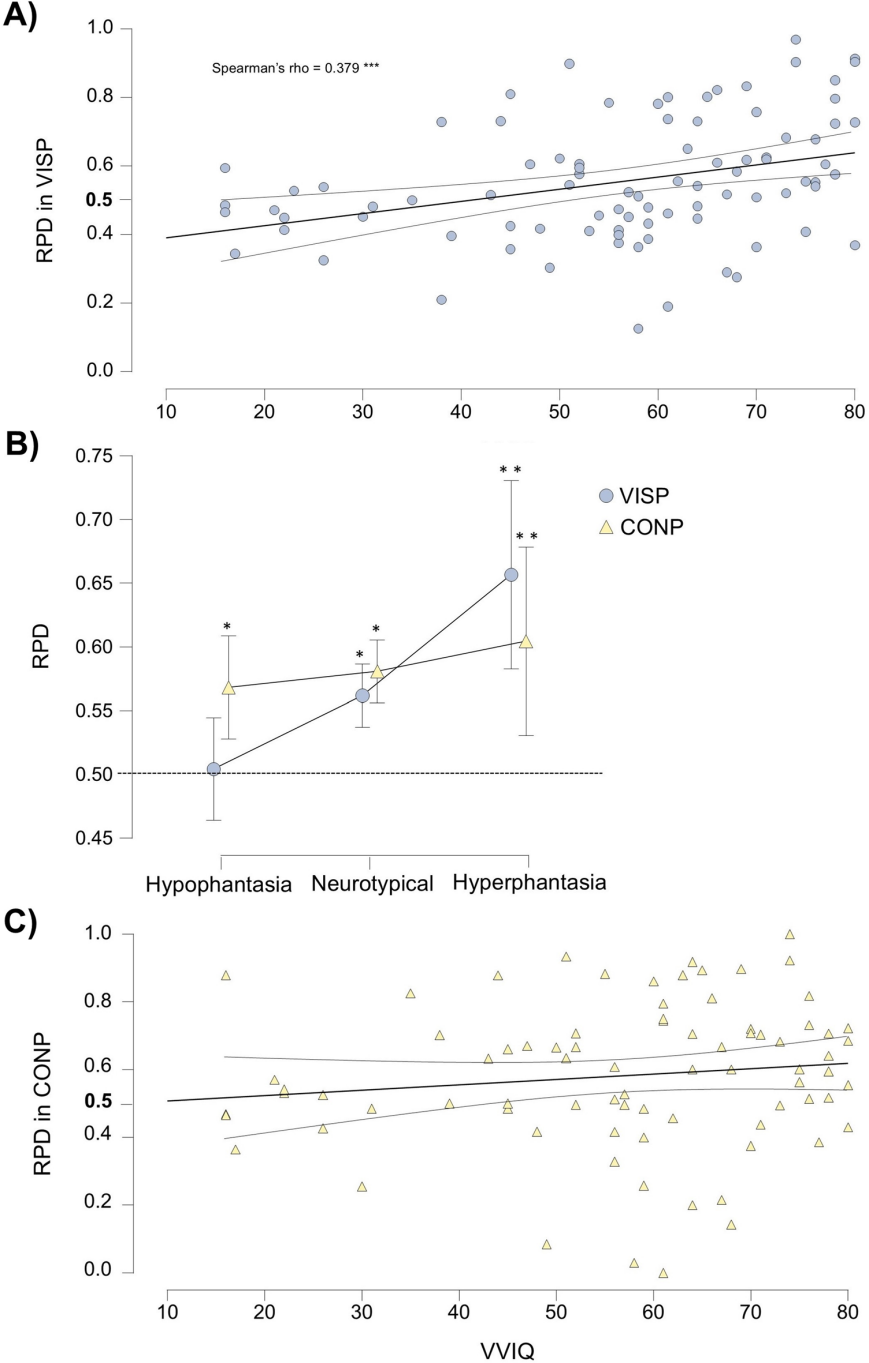
Modulatory effect of Internally-Generated Priming across the self-reported imagery vividness spectrum. (**a**) Correlation between the Ratio of Prime Direction (RPD) and Vividness of Visual Imagery Questionnaire (VVIQ) scores in the Visual Imagery Priming (VISP) condition with the fitted regression line and the 95% confidence interval. RPD expresses the direction and strength of perceptual modulation, where 0.5 indicates the absence of modulation. (**b**) RPD averages for the VVIQ assessed imagery vividness groups for the VISP and Conceptual Priming (CONP) conditions, with 95% confidence intervals. Asterisks indicate significance levels for one sided one-sample t-tests against 0.5 (*p < 0.05, **p < 0.01). (**c**) Correlation between RPD and VVIQ scores in the CONP condition, with the fitted regression line and the 95% confidence interval.

When analyzing the entire VVIQ scale, a significant positive correlation was found between RPD and VVIQ scores in the VISP condition (Spearman’s rho = 0.379, *p* < 0.001) (Fig. 3a). However, no such correlation was observed in the CONP (Spearman’s rho = 0.174, *p* = 0.124) (Fig. 3c) or SDP (Spearman’s rho = − 0.004, *p* = 0.969) conditions. To further assess the strength of evidence for or against the presence of a correlation in the non-significant conditions, we computed Bayes factors (BF_10_) for the correlations using Kendall’s Tau B. These analyses revealed strong evidence for a correlation in the VISP condition (Tau B = 0.252, BF_10_ = 32.588), but provide anecdotal-to-substantial evidence supporting the absence of correlation in the CONP (Tau B = 0.104, BF_10_ = 0.362) and SDP conditions (Tau B = 0.004, BF_10_ = 0.146), indicating that the non-significant results in these latter conditions are more likely to reflect genuine absence of effect rather than insufficient data sensitivity^25^.

### Perceptual modulation within imagery groups

For further analysis, participants were categorized into three imagery groups based on their VVIQ scores: hypophantasia (VVIQ < 48; n = 22), neurotypical imagery (48 ≤ VVIQ < 75; n = 53), and hyperphantasia (VVIQ ≥ 75; n = 14) (see Methods/Participants). The cutoff scores were based on Gulyás et al. (2022)^26^; however, in our analysis, we merged the aphantasia and hypophantasia ranges into a single “hypophantasia” group due to the low number of participants scoring in the aphantasic range. According to the Shapiro–Wilk test, all three groups followed a normal distribution across all conditions.

To determine whether the modulation effects were significant, one-sided one-sample t-tests were conducted, testing the RPDs against the baseline (no bias) 0.5 value (Fig. 3b). In the VISP condition, the hypophantasia group did not show significantly greater RPD than 0.5 (t(21) = − 0.272, *p* = 0.606, one-tailed, M = 0.492, SD = 0.140; BF_10_ = 0.184), whereas both the neurotypical imagery (t(51) = 2.092, *p* = 0.021 one-tailed, Cohen’s d = 0.290, M = 0.554, SD = 0.186; BF_10_ = 2.187) and the hyperphantasia groups (t(13) = 3.419, *p* = 0.002, one-tailed, Cohen’s d = 0.914, M = 0.657, SD = 0.171; BF_10_ = 21.599) exhibited significantly increased RPDs. In the CONP condition, all three groups demonstrated RPD values significantly greater than 0.5 (hypophantasia: t(19) = 1.868, *p* = 0.039, one-tailed, Cohen’s d = 0.418, M = 0.568, SD = 0.163; BF_10_ = 1.894; neurotypical imagery: t(45) = 2.126, *p* = 0.020, one-tailed, Cohen’s d = 0.313, M = 0.581, SD = 0.258; BF_10_ = 2.417; hyperphantasia: t(13) = 3.21, *p* = 0.003, one-tailed, Cohen’s d = 0.859, M = 0.604, SD = 0.122; BF_10_ = 15.535). Finally, in the SDP condition, RPD values were significantly lower than 0.5 across all groups (hypophantasia: t(21) = − 3.520, *p* = 0.001, one-tailed, Cohen’s d = − 0.750, M = 0.399, SD = 0.134; BF_10_ = 38.528; neurotypical imagery: t(52) = − 3.918, p < 0.001, one-tailed, Cohen’s d = − 0.538, M = 0.409, SD = 0.168; BF_10_ = 187.447; hyperphantasia: t(13) = − 2.614, *p* = 0.011, one-tailed, Cohen’s d = − 0.699, M = 0.350, SD = 0.215; BF_10_ = 6.029).

### Priming condition and imagery group interaction

We conducted a (2 × 3) mixed ANOVA with condition (VISP, CONP) as a within-subject factor and imagery group as a between-subject factor. Due to the repeated measures design, missing values (1 VISP neurotypical, 2 CONP hypophantasics, 7 CONP neurotypical) were replaced with the condition mean to maintain sample size. Missing data resulted from eye-tracking failure, absence of detectable nystagmus, there were fewer than four trials in the given condition where the proportion of mixed percepts was below 70%. The analysis revealed no significant main effect of condition (F(1, 84) = 0.572, *p* = 0.451) or imagery group (F(2, 84) = 1.143, *p* = 0.324), but a significant interaction between condition and imagery group (F(2, 84) = 3.417, *p* = 0.037, partial η^2^ = 0.009).

### Group differences within priming conditions

**Fig. 4:**
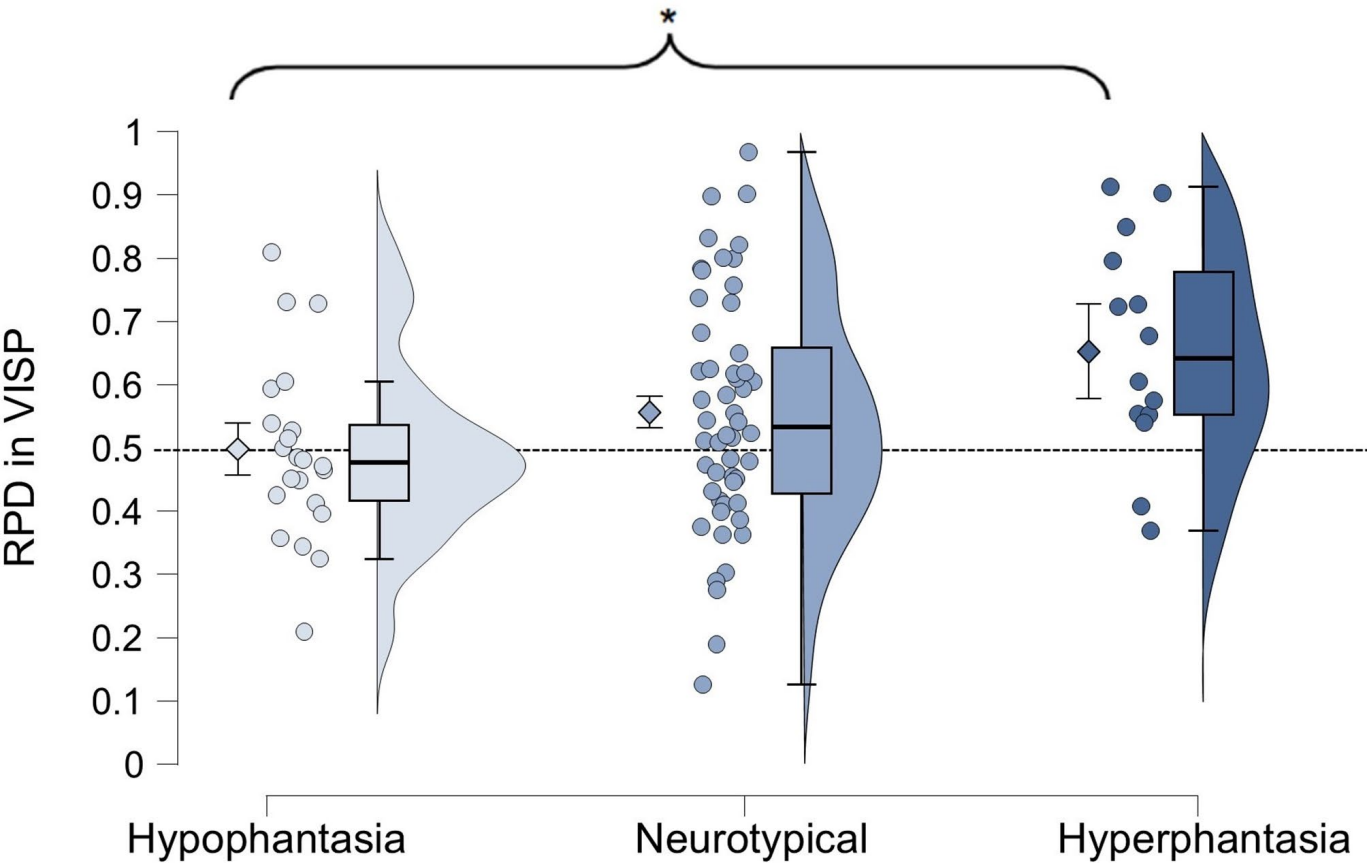
Modulatory effect of Visual Imagery Priming (VISP) across different self-reported imagery vividness groups. Ratio of Prime Direction (RPD) expresses the direction and strength of perceptual modulation, where 0.5 indicates the absence of modulation. The asterisk indicates significant difference (*p* = 0.018) between the hypo- and hyperphantasia groups. Diamonds represent group means with 95% confidence intervals, dots show individual data points, the box plots display the medians and quartiles, and violin plots illustrate data distribution.

Post-hoc analyses examined group differences within each condition. In the VISP condition, a one-way ANOVA revealed a significant effect of imagery group (F(2, 86) = 3.901, *p* = 0.024, η^2^ = 0.083). Post-hoc Tukey tests indicated a significant difference between the hypophantasia and hyperphantasia groups (*p* = 0.018, Cohen’s d = 0.955), whereas no significant difference was found between the hypophantasia and neurotypical imagery groups (*p* = 0.335) or between the neurotypical imagery and hyperphantasia groups (*p* = 0.124) (Fig. 4). In the CONP condition, a Kruskal–Wallis test (used due to significant Levene’s test for variance heterogeneity, *p* = 0.032) showed no significant differences among groups (*p* = 0.598).

### Effect of internally-generated priming in the hypophantasia group

**Fig. 5:**
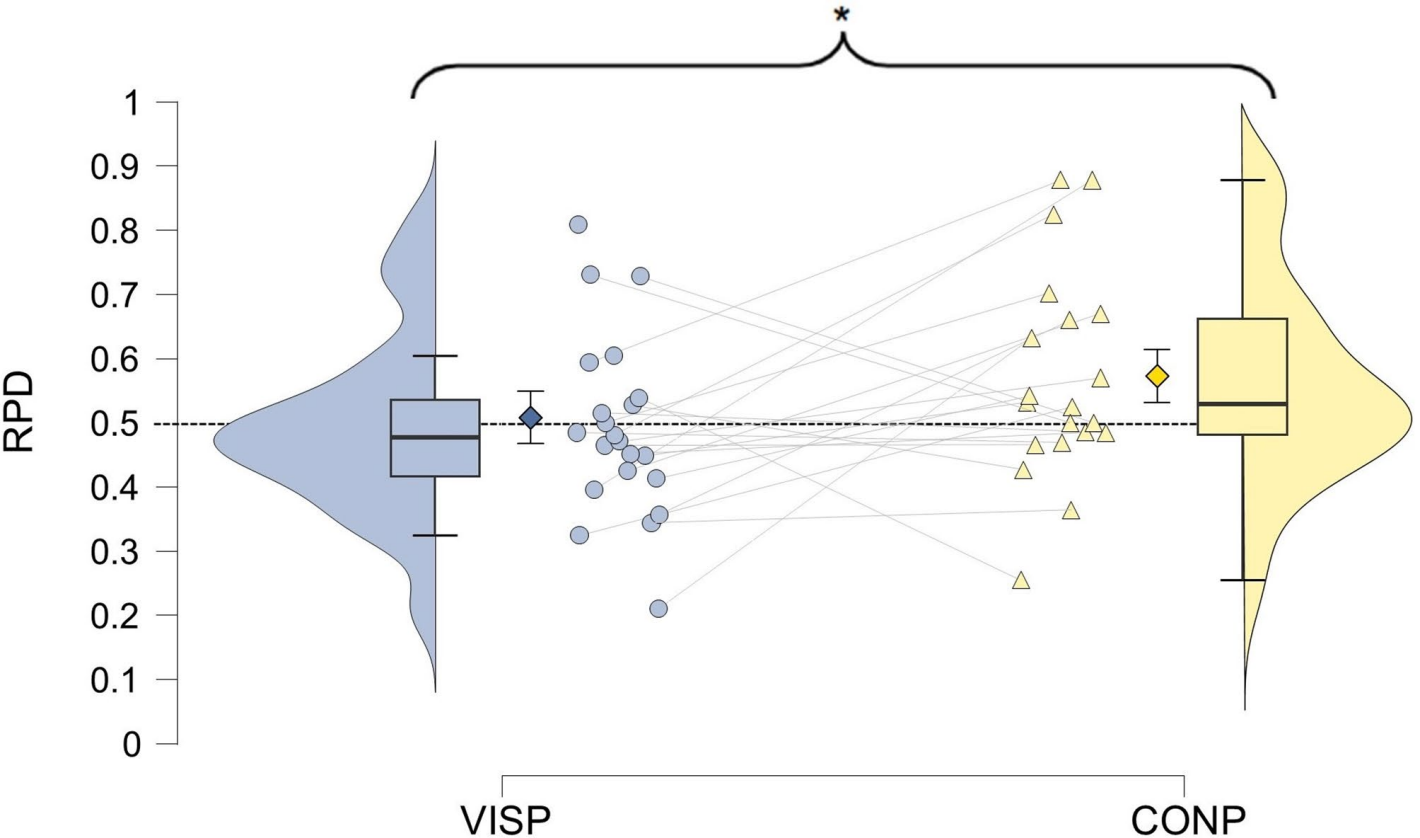
Effect of Internally-Generated Priming in the Hypophantasia Group. Ratio of Prime Direction (RPD) expresses the direction and strength of perceptual modulation, where 0.5 indicates the absence of modulation. Conceptual Priming (CONP) exhibited a significantly greater modulatory effect than Visual Imagery Priming (VISP) as indicated by the asterisk (*p* = 0.024). Diamonds represent group means with 95% confidence intervals, dots show individual data points with the lines connecting VISP and CONP RPD values of the same observers, box plots display the medians and quartiles, and violin plots illustrate data distributions.

Within-group comparisons using repeated measures ANOVA revealed that the hypophantasia group exhibited a significantly lower RPD in the VISP condition compared to the CONP condition (F(1, 20) = 5.994, *p* = 0.024, η^2^ = 0.231) (Fig. 5), whereas no significant condition differences were observed in the neurotypical imagery (F(1, 51) = 2.403, *p* = 0.127) or hyperphantasia (F(1, 13) = 1.165, *p* = 0.300) groups.

## Discussion

Mental simulation, the ability to internally model sensory, conceptual, or future events, is a crucial yet underexplored cognitive function. On the other hand, an important potential component of mental simulation, visual mental imagery, has been studied extensively relying on self-reports that are usually prone to bias. Our work addresses this limitation by introducing a no-report version of binocular rivalry priming, leveraging eye-tracking-based optokinetic nystagmus (BR-OKN) for a more objective measure of perceptual shifts. Additionally, we proposed conceptual priming as a novel approach to investigate abstract representations in mental simulation, providing new insights into alternative cognitive pathways beyond sensory-based imagery.

**Fig. 6:**
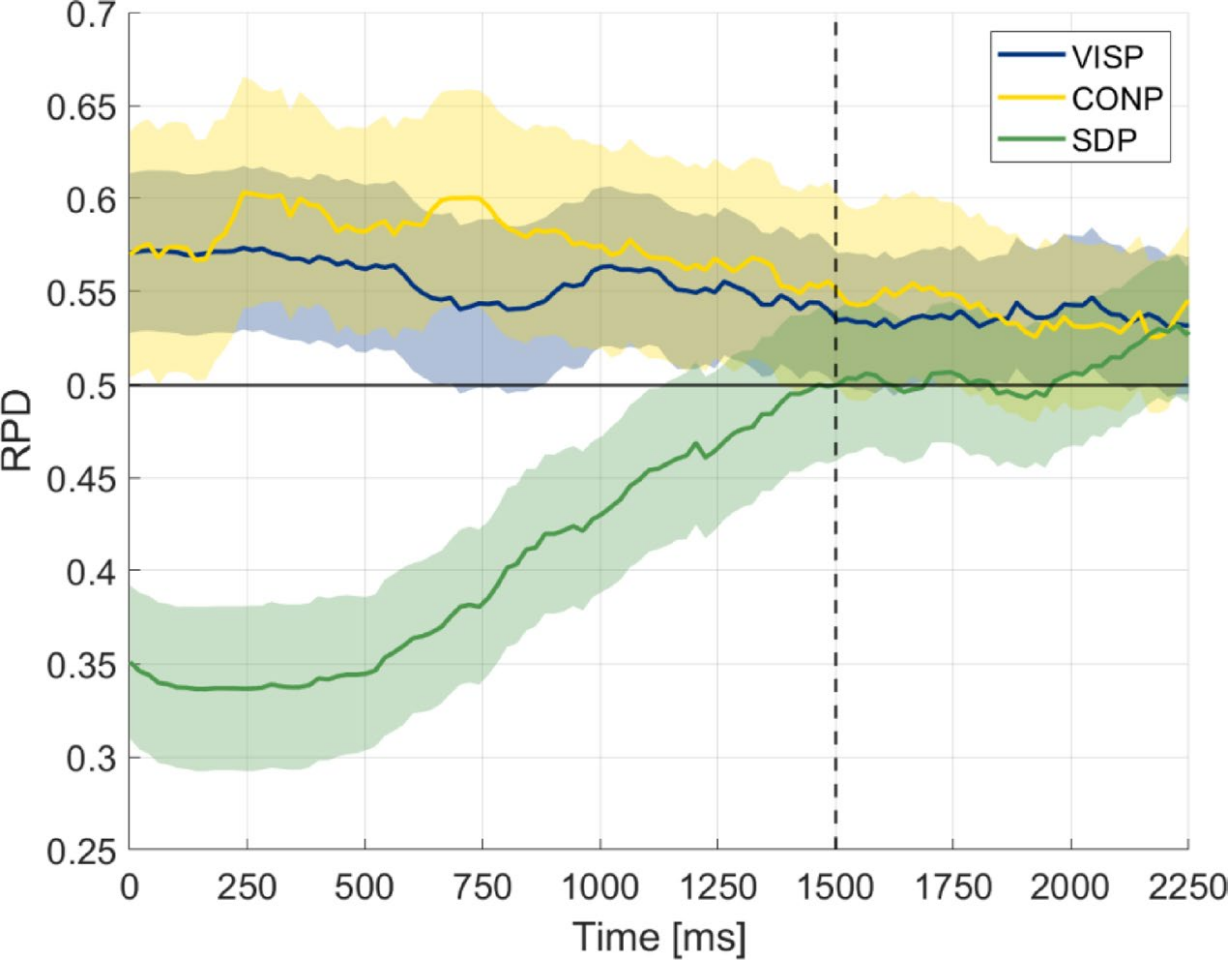
Temporal dynamics of priming. The x-axis shows the sequence of unmixed perception (left or right) periods after removing mixed perception phases. The curves show a moving average of the Ratio of Prime Direction (RPD), which expresses the direction and strength of perceptual modulation, where 0.5 indicates the absence of modulation. The moving average was calculated using a 10 ms time window with an 8 ms overlap. The blue, yellow, and green lines represent Visual Imagery Priming (VISP), Conceptual Priming (CONP), and Stimuli-Driven Priming (SDP), respectively. The shaded regions indicate the 95% confidence interval of the mean.

We found a consistent negative priming (adaptation) effect in the Stimulus-Driven Priming (SDP) condition across the entire spectrum of self-reported visual imagery vividness which is in line with other studies employing similar conditions^6,20^. This result indicates that our participant pool was homogenous in terms of sensitivity to priming under binocular rivalry conditions. Additionally, it allowed us to define the 1.5-s time window within which modulatory effects are likely to occur in our paradigm (see Methods/Fig. 6), thereby providing a useful parameter for further analyses.

In the Visual Imagery Priming (VISP) condition we found a significant correlation between self-reported imagery vividness and perceptual modulation under binocular rivalry (Fig. 3a). Further, while neurotypical imagery or hyperphantasic participants exhibited significant visual imagery priming, participants with hypophantasia did not show such modulation (Figs. 3b, 4).

In contrast, Conceptual Priming (CONP) showed no significant correlation between self-reported imagery vividness scores and perceptual modulation under binocular rivalry (Fig. 3c). However, supporting our hypothesis that alternative routes may enable internal representation activation, allowing for a more abstract strategy in internally-generated priming, we found significant modulatory effects across the entire imagery vividness spectrum, including at the hypophantasic end (Figs. 3b, 5).

Our hypophantasic participants clearly benefited from conceptual priming but not from visual imagery priming (Figs. 3b, 5). For this group, visual imagery was ineffective, whereas conceptual priming induced a clear modulatory effect. However, it is important to note that even the conceptual priming effect in hypophantasics was relatively modest, especially when compared to the strong imagery-based priming observed in hyperphantasics. Among participants with neurotypical imagery, the two types of internally-generated priming conditions produced comparable modulatory effects (Fig. 3b), which may be explained by the inherent uncertainty of self-report measures in ranking mid-range imagery vividness, or by the inability of participants to suppress visual imagery^27^.

Given that internal simulation in hyperphantasia may be more closely tied to sensory-perceptual representations, we initially hypothesized that conceptual priming might fail to modulate perception in this group. However, the observed success of conceptual priming could be attributed to two possible mechanisms: (1) hyperphantasic individuals might be adept at switching between different types of representations, or (2) they may struggle to inhibit visual imagery following the conceptual cue, inadvertently engaging in visual simulation even in the conceptual condition. Individual verbal reports of our hyperphantasic participants appear to be compatible with the second option. Nevertheless, the strength of the conceptual priming effect may also indicate a generally enhanced capacity for internal simulation across modalities in this group, a possibility that warrants further investigation.

To interpret these findings in the context of neurodevelopment and neurodiversity, a more integrative approach is needed for imagery research within neuroscience. This requires shifting the focus from considering mental images as degraded copies of perception within the visual system^14–18^ towards internally generated constructs that may emerge from neural networks largely decoupled from direct sensory input. One possibility is to consider the critical, and even causal role of the Default Mode Network (DMN) in perceptual alternations under BR^28^. These findings suggest that the internal dynamics between the DMN and the visual cortex serve as a “switch” in binocular rivalry, shaping the content of perceptual awareness. As the DMN is not part of the visual processing hierarchy, yet it seems to define the contents of visual perceptual awareness^28^, its role in both visual imagery and mental simulation might be hypothesized^29,30^, and future studies may attempt to broaden the perspective to include these interacting networks. In fact, according to a recent proposition, particular DMN structures responsible for interoception may also be related to voluntary mental imagery^31^. A heterarchical view on the neural architecture^32–34^ of visual imagery replacing the current, more hierarchical one^14–18^ has also been suggested based on extensive reviews of the scientific literature on visual mental imagery. The predictions of the dominant model considering visual imagery a reversed version of perception^14–18^ have been contradicted by findings clearly demonstrating the role of several sub-networks outside of the visual hierarchy^32–34^.

As our findings demonstrating conceptual priming in hypophantasic individuals also indicate that visual perceptual content in the presence of bistable perceptual input can be modulated by internal processes outside of the visual network, we believe that the current view assuming an exclusive link between visual imagery and sensory perception^5–7^ has been challenged. Based on that, we would like to suggest widening the perspective towards neurodiversity in mental simulations that may still include visual imagery, however, not as a mandatory feature, but as an optional asset that can be replaced by other qualities. Current research^1–4,16^ often categorises neurodivergent individuals as either “aphantasic” (lacking imagery) or “hyperphantasic” (experiencing extremely vivid imagery). We would like to suggest the introduction of a multidimensional spectrum rather than a binary classification. Individual differences likely reflect variations in the functional connectivity of heterarchical cortical networks, rather than the simple presence or absence of imagery within the visual hierarchy^32–34^. In line with a neuroaffirmative perspective, we propose a reframing of traditional terms describing individual differences in mental imagery vividness. In this framework, individuals with minimal reliance on sensory imagery are described as demonstrating an abstract simulation style, those with less vivid imagery as employing a low-sensory simulation style, and those with highly vivid imagery as engaging in high-sensory simulation style. This terminology reflects the recognition that all simulation styles offer distinct cognitive strengths and contribute uniquely to problem-solving, creativity, and adaptive thinking. By shifting the emphasis from absence or excess toward diversity in cognitive strategies, we aim to promote a more inclusive and nuanced understanding of mental imagery as one dimension of broader neurocognitive variation.

Among the limitations of our study is the reliance on a subjective questionnaire (VVIQ), which is prone to bias, interpretation variability, and demand characteristics within VVIQ extreme groups such as aphantasics^35^. To address this, future research could adopt implicit measures such as semi-structured interviews for group classification or explore several objective correlates replacing self-reports. Another limitation is the demographic composition of our sample, which primarily consisted of psychology students, potentially skewing VVIQ distributions and underrepresenting individuals with lower imagery vividness^26^. Future studies should recruit participants across a broader age range and use targeted pre-screening methods to ensure a more balanced representation. Furthermore, there was a gender imbalance in the sample, with a predominance of female participants. Although we have not found any gender-related effects (see Supplementary Material), future studies should aim for a more balanced gender distribution or perhaps explicitly investigate gender-related differences. It is also for future studies employing cortical imaging in addition to behavioural measures to investigate whether involuntary unconscious imagery^27,36,37^ played a role in modulating perceptual alternations under binocular rivalry. It might also be an interesting prospect in the future to involve more complex stimuli in the priming paradigm to dissociate further subprocesses of mental imagery and mental simulation.

In conclusion, our findings reinforce the notion that visual imagery as one of the human mental simulation abilities is a highly individualised cognitive function, potentially shaped by the intrinsic connectivity between the visual system and the associative network. Rather than viewing hypophantasia and hyperphantasia as suppressed or boosted activity within the visual system, we propose that they reflect distinct but equally valid neural strategies for engaging with imagined content and simulating future events. This perspective aligns with the broader neurodiversity framework, where cognitive variability is seen not as impairment but as a fundamental feature of human thought. To move toward a more neuroaffirmative perspective, we propose that the term “aphantasia”, which implies an absence of imagery, be reframed as "abstract simulation style," highlighting the strengths of non-visual cognitive strategies rather than positioning them as deficits.

## Methods

### Participants

A total of 119 participants were recruited for this study using convenience sampling, consisting of 94 university students who received extra credit for their participation, 7 individuals who have participated in an earlier experiment and were re-invited, and an additional 18 individuals were recruited using the snowball technique. After the exclusions (detailed below) 89 participants remained for the calculations presented here.

Participants were excluded if they lacked stereoscopic vision, confirmed through pre-screening^38^, and additional exclusion criteria included previously diagnosed neurodegenerative diseases, chronic neurological, neurodevelopmental or psychiatric conditions impacting cognition, and a history of severe head trauma with potential long-term cognitive effects. Based on these criteria, 9 participants were excluded. The mean age of participants was 21.9 years (SD = 3.97), and 82.7% of the participants were female.

Sample size was determined via power analyses for correlational (ρ = 0.4, power = 0.90, α = 0.05, required n ≈ 59) and one-sample t-test analyses (d = 0.5, power = 0.90, α = 0.05, required n ≈ 44). While the overall sample size was sufficient, group sizes based on imagery strength were unequal (hypophantasia: n = 22, typical imagery: n = 53, hyperphantasia: n = 14), and the smaller samples in the extreme groups may have reduced statistical power for some comparisons.

An Ocular Dominance Index (ODI) was calculated using the formula $ODI = \left|\frac{tright -tleft }{tright+tleft}\right|$, where t_left_ and t_right_ represent the time each eye was dominant^39^. 6 participants had less than 3 valid baseline trials (less than 70% mixed percepts), making it impossible to calculate their eye dominance reliably. Additionally, for 15 participants, ODI exceeded the pre-established 0.25 threshold. These participants were also excluded from the analysis.

Ethical approval for this study was provided by the Ethical Committee of Pázmány Péter Catholic University (PPCU), Budapest, Hungary. All experiments were performed in accordance with relevant guidelines and regulations.

### Assessment of imagery

To assess visual imagery vividness, we administered the Hungarian version of the VVIQ^40^, which was translated by our research group. VVIQ prompts participants to imagine specific scenarios—such as a close friend, a sunrise, a shop, and a rural scene—and to evaluate the vividness of four details for each scene. Participants rated their mental imagery on a scale following the approach of Zeman^1,3^ with responses ranging from 1 ("No image at all; you only know that you are thinking of the object") to 5 ("Perfectly clear and as vivid as real seeing"). The total VVIQ score was calculated by summing the individual item scores, yielding a range from 16 (indicating low imagery vividness) to 80 (indicating high vividness).

The average VVIQ for women aged 20–30 is expected to be around 54^26^, and since the younger portion of this age group constitutes the majority of our sample, the average VVIQ of 57 (SD = 17) aligns with this expectation. Participants were divided into three groups based on their VVIQ scores, following the thresholds outlined by Gulyas^41^. The aphantasia group (VVIQ ≤ 19) included only 4 participants, so it was merged with the hypophantasia group, resulting in a combined group with VVIQ scores of 47 or below (22 participants). The neurotypical imagery group consisted of participants with VVIQ scores between 48 and 74 (53 participants). Finally, the hyperphantasia group comprised individuals with exceptionally vivid imagery, scoring 75 or above (14 participants). This categorization ensured representation across the full spectrum of imagery vividness.

### Experimental setup, paradigm and stimulus parameters

The experimental setup followed the design developed by our group earlier^23,24^, using two monitors, a mirror stereoscope, and an eye-tracking device to measure eye movements (Fig. 1). This arrangement allowed for accurate control and observation of the perceptual experiences of participants.

Participants viewed the visual stimuli through a dichoptic setup involving two LCD displays, with a 0.26 cycles per degree spatial frequency 48 pix/° spatial resolution and a refresh rate of 120 Hz. Participants viewed these displays through a mirror stereoscope attached to a headrest, with cold mirrors. Eye movements were tracked using an EyeLink 1000 Plus eye-tracking system to ensure precise measurement of gaze behavior^41^. Gratings moved horizontally in both monitors either in the same direction (cue) or in opposite directions (for perceptual rivalry). Each grating covered a rectangular viewing area of 15.2° in width by 8.4° in height. The stimulus contrast was 50% and the temporal frequency was 8.7 cycles/s, with a motion speed of 33.5°/s or 1600 pixels/s. Each grating was framed within a rectangular box with a random texture pattern to facilitate binocular fusion. All stimuli were generated using MATLAB R2019b^42^ and the Psychophysics Toolbox^43^.

Upon arrival, participants completed an informed consent form, a health declaration, and provided demographic information, including age, gender, and diopter prescription. We also assessed their stereoscopic vision using the Super Stereoacuity Timed Tester^38^.

Before the main experiment, participants completed a practice trial, during which they were informed about the binocular rivalry task. They were instructed to view the display attentively and to follow the horizontally moving gratings with their gaze, using the analogy of following passing trees from a moving train to help them focus. Participants were not required to report their perception of the stimuli, as perceptual states and transitions were later inferred from eye-movement recordings.

As illustrated in Fig. 2, the experimental paradigm had two types of Internally-Generated Priming blocks: Visual Imagery Priming (VISP) and a Conceptual Priming (CONP). In a third block, the Stimulus-Driven Priming (SDP) rivalry stimulus replaced the imagery window. Each block consisted of two 25-s baseline rivalry sessions followed by 12 priming trials, with directions alternating randomly between leftward and rightward (in equal proportion) within each block.

Each priming trial included three phases. First, a 2-s cue was presented identically to both eyes. This was followed by a 5-s imagery phase, which was displayed as a grey rectangle, or, in the case of SDP, the continuation of the cue without a break. Finally, a 10-s binocular rivalry phase occurred, which was subsequently analysed in further detail.

For the VISP block, participants were instructed to imagine the direction of the nonrivalrous cue as a picture or video during the duration of the grey rectangle. In the CONP block, participants were instructed to think about and silently repeat the cued direction, e.g., 'right, right, right…', for the duration of the grey rectangle (see the complete set of instructions in the Supplementary Material). As shown in Fig. 6, inhibitory effects of the cue typically dissipate within approximately 1.5 s. The 5-s imagery/conceptual phase was therefore considered sufficient to eliminate any direct influence of the cue on subsequent binocular rivalry. During the rivalry phase, there was no task other than attending to the presented stimulus.

### Data collection and analysis

When a competitive visual stimulus triggers horizontal optokinetic nystagmus (OKN), the direction of smooth pursuit phases provides a reflexive response indicating the participant’s perceived direction of motion. During the experiment, we tracked the horizontal eye movements of participants at a sampling rate of 1000 Hz and analyzed shifts in perceived direction using the cumulative smooth pursuit (CSP) method, as outlined in our previous study^24^. This approach first filters out invalid data points (such as those caused by blinks or other artifacts) and isolates slow pursuit segments based on a combination of criteria: low velocity, low acceleration, and a minimum duration^24^. Once these segments are identified, the method aligns them, fills in any gaps through interpolation, and resamples the data multiple times using a “bagging” technique. This procedure generates an estimated eye velocity profile, which includes median values and 95% confidence intervals for each time point. Dominance periods are defined as continuous intervals where the entire 95% confidence interval consistently stays above or below a defined gaze velocity threshold. Any remaining periods are categorized as perceptual transition intervals, or mixed percept^24^.

The Ratio of Prime Direction (RPD) was calculated as the proportion of eye movements in the direction of the primed grating within the first 1500 ms of unmixed perception (Fig. 6.). This 1500 ms interval was chosen, because the confidence intervals of RPD in all three priming conditions at 1500 ms already encompass 0.5, indicating that the modulation is reduced, the RPD is returning back to baseline. We verified that the RPD value is a reliable indicator across all three conditions (see Supplementary Material). The RPD measure is also reliable in terms of indicating the perceived direction of movement under BR as the OKN response can only be elicited by actual stimulation, not by internally generated processes. The direction of slow vs. fast phases of OKN expresses the stimulus movement direction that the participant is aware of under bistable stimulation^22–24^.

## Electronic supplementary material

Below is the link to the electronic supplementary material.

Supplementary Material 1

## Data Availability

The datasets used in this study and Supplementary Material are available at the Open Science Framework (OSF) platform at the following address: https://osf.io/mzb5j/?view_only=5841afc4320f418d8c9b59177df56219.

## Abbreviations

BR: Binocular rivalry
CONP: Conceptual priming
OKN: Optokinetic nystagmus
SDP: Stimulus driven priming
VISP: Visual imagery priming
VVIQ: Vividness of visual imagery questionnaire
