# Supplementary material for: Neurodiversity in mental simulation: conceptual but not visual imagery priming modulates perception across the imagery vividness spectrum
Source: Sci Rep. 2025 Jul 1;15:22217. doi: 10.1038/s41598-025-05100-2 (PMC12216594; doi:10.1038/s41598-025-05100-2)
Supplement: Supplementary file 1 — Supplementary Material 1 [file 41598_2025_5100_MOESM1_ESM.pdf]

Supplementary Material for

Neurodiversity in Mental Simulation: Conceptual but Not Visual Imagery Priming Modulates Perception Across the Imagery Vividness Spectrum

Ágnes Welker[1,2], Orsolya Pető-Plaszkó[1], Luca Verebélyi[1], Ferenc Gombos[3,4], István Winkler[1], Ilona Kovács[4,5]

[1] HUN-REN Research Centre for Natural Sciences, Budapest, Hungary

[2] Semmelweis University Doctoral School, Budapest, Hungary

[3] Pázmány Péter Catholic University, Budapest, Hungary

[4] HUN-REN-ELTE-PPKE Adolescent Development Research Group, Budapest, Hungary

[5] Eötvös Loránd University Faculty of Education and Psychology, Budapest, Hungary

Our data can be accessed via this link: https://osf.io/mzb5j/?view_only=5841afc4320f418d8c9b59177df56219

Statistical tests were conducted using JASP software (JASP Team, 2024. JASP (Version 0.19.0)). This document includes the analyses of the RPD (Ratio of Prime Direction) within the first 1500 ms of unmixed perception. Stars indicate the significance level: * $p < .05$, ** $p < .01$, *** $p < .001$.

For further details, see the Methods section.

One sided one sample t-test

		t	df	p	Cohen's d	SE Cohen's d
VISP	H1: >0.5	2.868	87	0.003**	0.306	0.109
CONP	H1: >0.5	3.380	79	<.001***	0.378	0.116
SDP	H1: <0.5	-5.745	88	<.001***	-0.609	0.115

Supplementary Table 1: One sided one sample t-tests for each condition.

Three conditions were measured in this article: Visual Imagery Priming (VISP), Conceptual Priming (CONP), and Stimulus Driven Priming (SDP). Notably, each condition has a priming effect, with the strongest observed effect in SDP condition. Both in VISP ($t(87) = 2.868$, $p = 0.003$, one-tailed, Cohen's d = 0.306) and in CONP ($t(79) = 3.380$, $p < 0.001$, one-tailed, Cohen's d = 0.378) there is a positive, but in SDP ($t(88) = -5.745$, $p < 0.001$, one-tailed, Cohen's d = -0.609) there is a negative priming effect.

VVIQ

Table 1 Descriptive statistics

	VVIQ
N	89
Missing	0
Median	59.000
Mean	56.506
Std. Deviation	17.521
Shapiro-Wilk	0.921
P-value of Shapiro-Wilk	< .001***
Minimum	16
Maximum	80

Supplementary Table 2: Descriptive statistics of VVIQ.

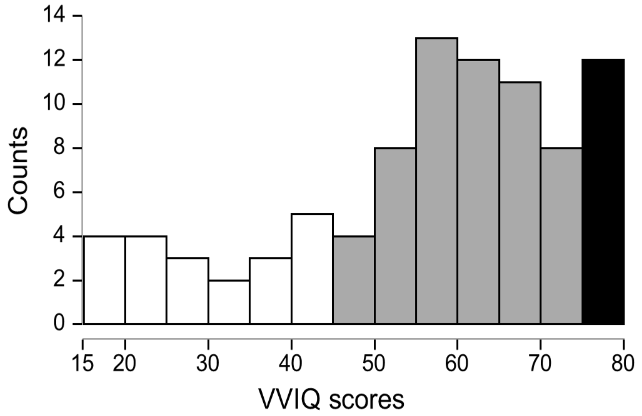

Supplementary Figure 1: Histogram of the VVIQ, colors indicate the imagery groups.

Group formation based on Vividness of Visual Imagery (VVIQ) Scores: Participants were divided into three groups based on their VVIQ scores. The hypophantasia group included individuals with VVIQ scores 47 or below, the hyperphantasia group included those with scores 75 or higher, while the remaining participants were classified into the typical imagery group.

Descriptive Statistics of Ratio of Prime Direction (RPD)

		Valid	Missing	Median	Mean	Std. Deviation	Shapiro-Wilk	P-value of Shapiro-Wilk	Min	Max
VISP	Hypophantasia	22	0	0.476	0.492	0.140	0.959	0.469	0.210	0.809
	Neurotypical	52	1	0.532	0.554	0.186	0.986	0.796	0.126	0.968
	Hyperphantasia	14	0	0.641	0.657	0.171	0.955	0.644	0.369	0.913
CONP	Hypophantasia	20	2	0.528	0.568	0.163	0.942	0.264	0.255	0.878
	Neurotypical	46	7	0.621	0.581	0.258	0.959	0.105	0.000	1.000
	Hyperphantasia	14	0	0.598	0.604	0.122	0.981	0.983	0.386	0.818
SDP	Hypophantasia	22	0	0.392	0.399	0.134	0.984	0.962	0.152	0.681
	Neurotypical	53	0	0.391	0.409	0.168	0.986	0.794	0.000	0.770
	Hyperphantasia	14	0	0.304	0.350	0.215	0.954	0.625	0.028	0.800

Supplementary Table 3: Descriptive statistics for RPD in VISP, CONP and SDP conditions.

According to the Shapiro-Wilk test, all of them are normally distributed within each group. It is important to note that in the CONP condition, two data points are missing for hypophantasia, and seven for typical imagery. Additionally, in the VISP condition, one data point is missing for typical imagery.

Spearman's correlations

	Spearman's rho	p	Effect size (Fisher's z)	SE Effect size	Kendall's Tau B	BF(10)
**VVIQ ~ VISP**	0.379***	<.001	0.399	0.111	0.252	32.588
**VVIQ ~ CONP**	0.174	0.124	0.175	0.115	0.104	0.362
**VVIQ ~ SDP**	-0.004	0.969	-0.004	0.107	0.004	0.146

Spearman's correlation between VVIQ score and RPD

Supplementary Table 4: Spearman's correlation between VVIQ Scores and RPD in the different priming correlation.

While CONP and SDP do not correlate with VVIQ scores, VISP and VVIQ show a moderate correlation (Spearman's $\rho = 0.379$; Fisher's $z = 0.399$) with strong evidence ($p < 0.001$).

One sided one sample t-test

		t	df	p		Cohen's d	SE Cohen's d	BF(10)
VISP H1: >0.5	Hypophantasia	-0.272	21	0.606		-0.058	0.213	0.184
	Neurotypical	2.092	51	0.021	*	0.290	0.142	2.187
	Hyperphantasia	3.419	13	0.002	**	0.914	0.318	21.559
CONP H1: >0.5	Hypophantasia	1.868	19	0.039	*	0.418	0.233	1.894
	Neurotypical	2.126	45	0.020	*	0.313	0.151	2.417
	Hyperphantasia	3.21	13	0.003	**	0.859	0.313	15.535
SDP H1: <0.5	Hypophantasia	-3.520	21	0.001	**	-0.750	0.241	38.528
	Neurotypical	-3.918	52	<.001	***	-0.538	0.147	187.447
	Hyperphantasia	-2.614	13	0.011	*	-0.699	0.298	6.029

Supplementary Table 5: One-sided one-sample t-test comparing the sample mean to a reference value of 0.5.

As Supplementary Table 4 shows, VISP and CONP exhibit a significant positive deviation from 0.5, with stronger effects in hyperphantasia (in both cases, $p < 0.01$, one-tailed and Cohen's $d > 0.85$), except for hypophantasia in VISP ($p = 0.606$, one-tailed, $d = -0.058$). In SDP, all groups show a significant negative deviation from 0.5 ($p < 0.05$, one-tailed, in all cases).

Mixed ANOVA

Every group and condition has a normal distribution (see Supplementary Table 3). Due to the repeated measures design, missing values were replaced with the condition's mean to maintain sample size. Conditions refer to the VISP, CONP, and SDP conditions, and the imagery group refers to hypophantasic, neurotypical, and hyperphantasic groups.

Test for Equality of Variances (Levene's)

	F	df1	df2	p
VISP	1.896	2	84	0.156
CONP	3.592	2	84	0.032 *

Within Subjects Effects

Cases	Sum of Squares	df	Mean Square	F	p	η^2
Condition	0.005	1	0.005	0.572	0.451	0.001
Condition ✻ Imagery group	0.057	2	0.028	3.417	0.037 *	0.009
Residuals	0.699	84	0.008			

Between Subjects Effects

Cases	Sum of Squares	df	Mean Square	F	p	η^2
Imagery group	0.148	2	0.074	1.143	0.324	0.023
Residuals	5.455	84	0.065			

Supplementary Table 6: Mixed ANOVA analysis

There is a normal distribution in each condition (see Supplementary Table 3). Although Levene's test was significant in case of CONP ($F(2, 84) = 3.592$, $p = 0.032$), due to the largeness of the sample, mixed ANOVA is still enabled to use. Even though there is no significant main effect neither in the conditions ($F(1) = 0.572$, $p = 0.451$), nor in the imagery groups ($F(2) = 1.143$, $p = 0.451$), the interaction of the conditions and the imagery groups was significant with small effect size ($F(2) = 3.417$, $p = 0.037$, $\eta^2 = 0.009$).

Post-hoc tests for mixed ANOVA

Conditions refer to the VISP, CONP, and SDP conditions, and the imagery group refers to hypophantasic, neurotypical, and hyperphantasic groups.

ANOVA - VISP

Cases	Sum of Squares	df	Mean Square	F	p	η^2
Imagery group	0.232	2	0.116	3.901	0.024*	0.083
Residuals	2.562	86	0.030			

Post Hoc Comparisons - Imagery group

		Mean Difference	SE	t	Cohen's d	p_{tukey}	
Hypophantasia	Neurotypical	-0.062	0.044	-1.420	-0.360	0.335	
	Hyperphantasia	-0.165	0.059	-2.793	-0.955	0.018	*
Neurotypical	Hyperphantasia	-0.103	0.052	-1.979	-0.595	0.124	

Kruskal-Wallis Test - CONP

Factor	Statistic	df	p
Imagery group	1.027	2	0.598

Supplementary Table 7: Post-hoc test for mixed ANOVA

As the ANOVA analysis resulted in a significant effect on VISP in the imagery group ($F(2) = 3.901$, $p = 0.024$, $\eta^2 = 0.083$), post-hoc comparisons tests were used to observe which group caused the difference. Within all pairs of groups (hypophantasia - neurotypical, hypophantasia - hyperphantasia, neurotypical - hyperphantasia) only hypo- and hyperphantasia groups showed significant results. The mean difference was -0.165 (SE = 0.059), with a t-value of -2.793 and a Cohen's d of -0.955, showing a large effect size. The p-value from the Tukey test was $p = 0.018$, indicating that this difference is statistically significant. The Kruskal-Wallis test statistic was not significant ($p = 0.598$), which indicates no difference between the imagery groups in CONP condition.

Repeated measures ANOVA - Condition

	Cases	Sum of Squares	df	Mean Square	F	p		η^2
Hypophantasia	Condition	0.042	1	0.042	5.994	0.024	*	0.231
	Residuals	0.141	20	0.007				
Neurotypical	Condition	0.016	1	0.016	2.403	0.127		0.045
	Residuals	0.345	51	0.007				
Hyperphantasia	Condition	0.019	1	0.019	1.165	0.300		0.082
	Residuals	0.213	13	0.016				

Supplementary Table 8: Repeated measures ANOVA within conditions effects.

According to Supplementary Table 8, only the hypophantasic group had significantly different results as the condition changed ($F(1, 20) = 5.994$, $p = 0.024$, $\eta^2 = 0.231$). The other groups did not show significant differences in performance across conditions.

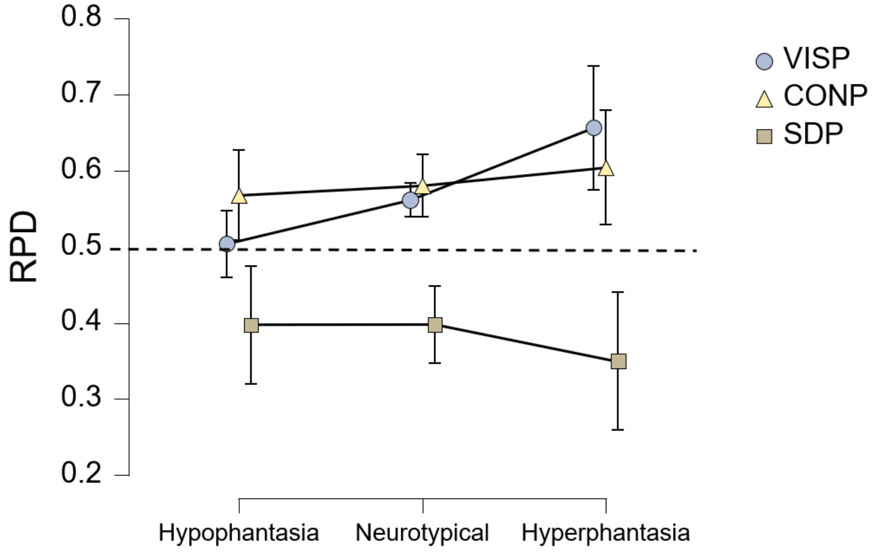

Supplementary Figure 2: Comparison of the VISP, PROP, and SDP conditions with the imaginary group resolution.

Reliability

The following includes the reliability analyses of the RPD (Ratio of Prime Direction) value for the visual imagery priming (VISP), stimulus driven priming (SDP) and conceptual priming (CONP) condition.

Descriptive statistics (N=48)

	VISP1	VISP2	CONP1	CONP2	SDP1	SDP2
Median	0.545	0.521	0.521	0.534	0.456	0.475
Mean	0.566	0.560	0.560	0.565	0.448	0.458
95% CI Mean Upper	0.622	0.611	0.611	0.615	0.501	0.513
95% CI Mean Lower	0.511	0.509	0.509	0.514	0.396	0.402
Std. Deviation	0.191	0.175	0.175	0.174	0.181	0.191
Shapiro-Wilk	0.974	0.954	0.954	0.966	0.985	0.991
P-value of Shapiro-Wilk	0.374	0.060	0.060	0.182	0.796	0.963
Minimum	0.189	0.036	0.036	0.224	0.000	0.025
Maximum	0.967	0.893	0.893	0.975	0.800	0.944

Supplementary Table 9: Descriptive statistics of the RPD in the different conditions and sessions.

With 48 subjects the whole paradigm was repeated after a 10 minute break, with an altered CONP, SDP, VISP order. Number 1 indicates the first session, number 2 the second one after the break.

Descriptive statistics (N=48)

	VISP1-VISP2	CONP1-CONP2	SDP1-SDP2
Median	0.020	0.005	-0.032
Mean	0.006	-0.005	-0.010
95% CI Mean Upper	0.062	0.034	0.028
95% CI Mean Lower	-0.049	-0.043	-0.047
Std. Deviation	0.191	0.134	0.129
Shapiro-Wilk	0.971	0.977	0.969
P-value of Shapiro-Wilk	0.277	0.473	0.221
Minimum	-0.325	-0.247	-0.250
Maximum	0.491	0.389	0.325

Supplementary Table 10: Descriptive statistics of the difference between the RPD value in the first and the second session by conditions.

We calculated the difference between the first and the second session for each condition.

One sample t-test

	**t(48)**	**p (2-tailed)**	**Cohen's d**	**SE Cohen's d**
**VISP1-VISP2**	0.231	0.819	0.033	0.144
**CONP1-CONP2**	-0.234	0.816	-0.034	0.144
**SDP1-SDP2**	-0.521	0.605	-0.075	0.145

Note: For the Student t-test, the alternative hypothesis specifies that the mean is different from 0.

Supplementary Table 11: One sample t-test on the difference variables.

The difference variables were examined using a one-sample t-test, revealing no significant difference from 0. This indicates that the first and second measurement occasions produced equal outputs.

Pearson's correlations between VISP1 and VISP2 (N=48)

Pearson's r	p	Effect size (Fisher's z)	SE Effect size
0.460***	< .001	0.498	0.149

Supplementary Table 12: Pearson correlation between first session's RPD and the second session's RPD in the VISP condition.

Pearson's test provides strong evidence for a moderate positive correlation between VISP1 and VISP2 (Pearson's r = 0.460, $p < 0.001$).

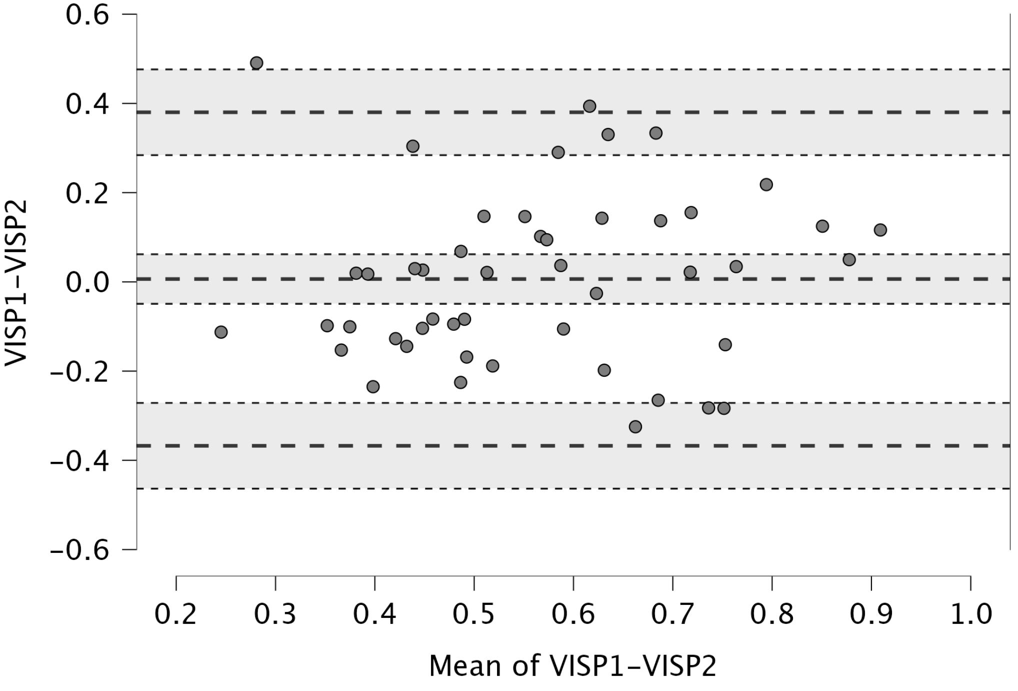

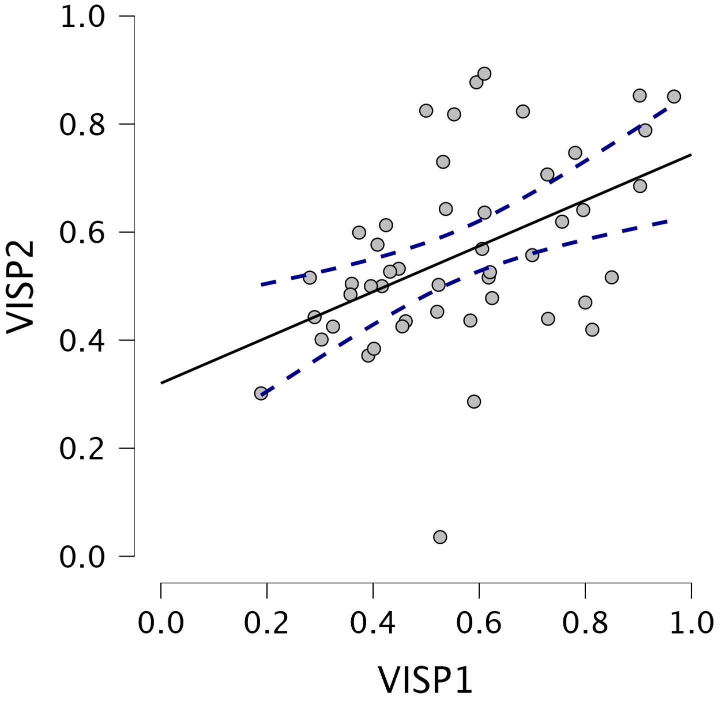

Supplementary Figure 2: Reliability of the VISP condition after a 10 minute break: Bland-Altman plot (left); correlation, the blue dashed line indicates the 95% confidence interval (right).

Pearson's correlations between CONP1 and CONP2 (N=48)

Pearson's r	p	Effect size (Fisher's z)	SE Effect size
0.706***	< .001	0.879	0.149

Supplementary Table 13: Pearson's correlation between first session's RPD and the second session's RPD in the CONP condition.

CONP1 and CONP2 are fairly strongly correlated (Pearson's r = 0.706, p < 0.001).

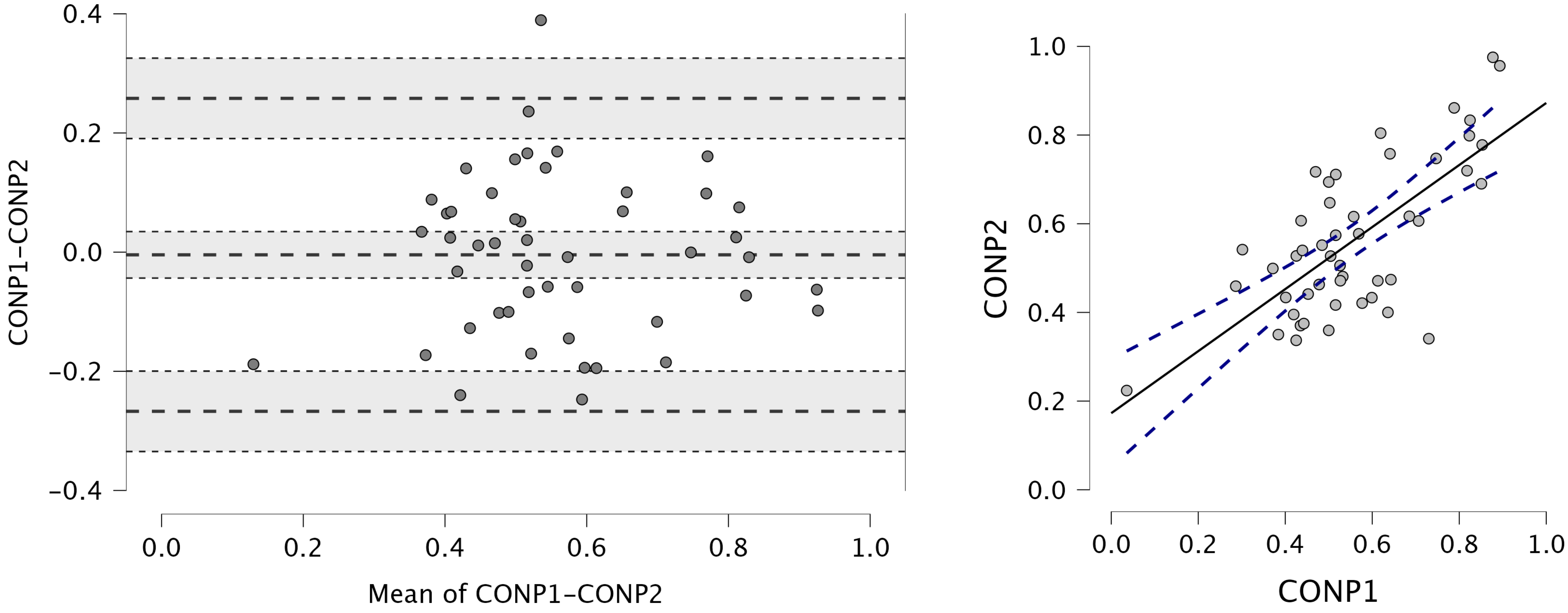

Supplementary Figure 3: Reliability of the CONP condition after a 10 minute break: Bland-Altman plot (left); correlation, the blue dashed line indicates the 95% confidence interval (right).

Pearson's correlations between SDP1 and SDP2 (N=48)

Pearson's r	p	Effect size (Fisher's z)	SE Effect size
0.759***	< .001	0.995	0.149

Supplementary Table 14: Pearson correlation between first session's RPD and the second session's RPD in the SDP condition.

SDP1 and SDP2 are strongly correlated (Pearson's r = 0.759, p < 0.001).

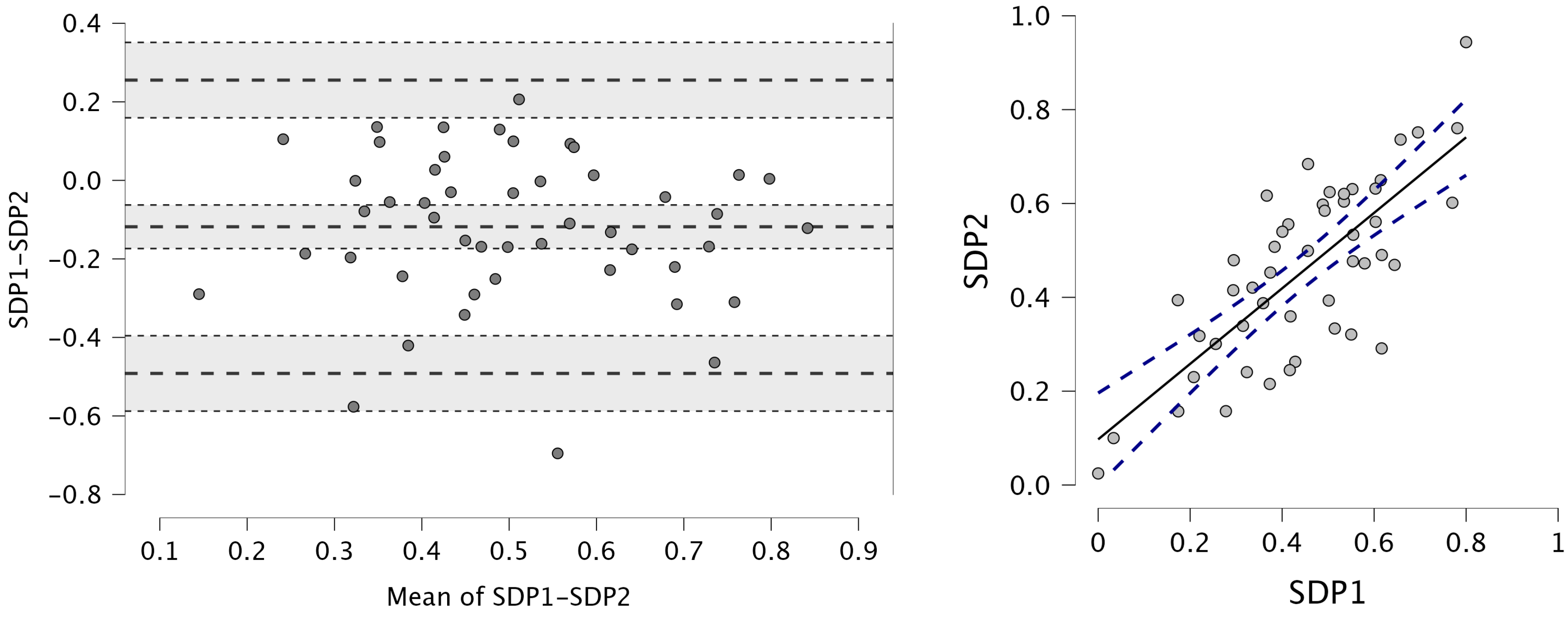

Supplementary Figure 4: Reliability of the SDP condition after a 10 minute break: Bland-Altman plot (left); correlation, the blue dashed line indicates the 95% confidence interval (right).

Sex differences

Test of normality (Shapiro-Wilk)

		W	p
VISP	female	0.979	0.262
	male	0.955	0.647
CONP	female	0.978	0.258
	male	0.917	0.262
SDP	female	0.994	0.971
	male	0.981	0.982

Note. Significant results suggest a deviation from normality.

Test of equality of variances (Levene's)

	**F**	**df_1**	**df_2**	**p**
VISP	2.617	1	86	0.109
CONP	0.544	1	78	0.463
SDP	0.723	1	87	0.398

Independent samples t-test

	**t**	**df**	**p**	**Cohen's d**	**SE Cohen's d**
VISP	0.102	86	0.919	0.030	0.291
CONP	-0.046	78	0.964	-0.014	0.313
SDP	-0.777	87	0.439	-0.226	0.292

Note. Student's t-test.

Supplementary Table 15: Sex differences within the conditions.

Firstly, the Shapiro-Wilk test confirmed normality, as no significant results were found within each condition and sex. Secondly, the non-significant Levene’s test indicates equal variances across conditions. Meeting these assumptions, the independent samples t-test revealed no significant differences within any group. This suggests that gender does not have a meaningful effect on the examined variables.

Instructions

The test will consist of 3 blocks, and each lasts about 5 minutes. We always calibrate at the beginning of the blocks, so you can take a break between blocks and move around, but during the experiment after calibration it is important to keep your head still.

Now I'm showing you what you're going to see, it's not part of the test yet.

Short presentation: basic rivalry + 1 imaginary block

Your two eyes both get a different view of the two monitors using the mirrors. The sinus grid moves in opposite directions. You will see the grid go in one direction and the other, and sometimes the two directions will mix. When the lanes are going in one direction, just let your eyes follow the stimulus, like watching trees go by while you're travelling. This eye movement is recorded with the eye movement tracker. Within the block, there are short sections of a few seconds, with a grey screen in between to indicate the end of the section. When this yellow dot appears, you can press enter to start the next section. So you can take a short break at this point as well, just don't move your head. But feel free to blink, you can wait a while.
When this grey rectangle appears, you will have a task, which I will tell you before each block.

Now please sit here, and adjust the height to make you comfortable for the next 20 minutes or so.

Visual Imagery Priming (VISP)
In the first block, after two basic rivalry sections, you will see a grid running in one direction (both eyes will then receive the same stimulus), followed by the grey rectangle in the middle of the screen shown at the beginning.
Now, for this block, when you see the grey rectangle, your task is to visualize the stimulus shown earlier (the grid running in one certain direction) onto.

Have you understood the task? Then place your chin here, rest your forehead here, look straight ahead. Before I start the calibration, find the enter with your hand, because you will be able to start the sections with it.
I'm starting the calibration now, you'll see a dot, just follow it with your eyes.
Okay, thank you. I'll start the test.

The first block is over, now you can move your head if you feel you need to take a break.

Stimulus Driven Priming (SDP)
The second block follows. Now all you have to do is follow the tracks with your eyes as if you were looking at the passing trees from a train window. We'll calibrate again. Place your chin here, rest your forehead here, look straight ahead. Before I start the calibration, find the enter with your hand.
I'm starting the calibration now, you'll see the dot again, just follow it with your eyes.
Okay, thank you. I'll start the test.

The second block is over, now you can move your head if you feel you need to take a break.

The third block follows. After the two basic rivalry sections, you will see a grid running clearly in one direction, followed by the grey rectangle in the middle of the screen that you know from the first block.

Conceptual Priming (CONP)
For this block, after you have seen the grids going in one direction and you see the grey rectangle, your task is to say the direction that you have seen before to yourself, for example, "right, right, right" or "left, left, left."

Have you understood the task? Then place your head, look straight ahead, find the enter with your hand, and we can start the calibration.
Okay, thank you. I'll start the test.

We are ready, thank you for coming!
